# Developmental and Epileptic Encephalopathy: Pathogenesis of Intellectual Disability Beyond Channelopathies

**DOI:** 10.3390/biom15010133

**Published:** 2025-01-15

**Authors:** Alexandra D. Medyanik, Polina E. Anisimova, Angelina O. Kustova, Victor S. Tarabykin, Elena V. Kondakova

**Affiliations:** 1 Institute of Neuroscience, Lobachevsky State University of Nizhny Novgorod, 23 Gagarin Ave., 603022 Nizhny Novgorod, Russia; al.medyanik111@gmail.com (A.D.M.); polina.adyasova@yandex.ru (P.E.A.); elakust@gmail.com (A.O.K.); elen_kondakova@list.ru (E.V.K.); 2 Institute of Cell Biology and Neurobiology, Charité—Universitätsmedizin Berlin, Charitéplatz 1, 10117 Berlin, Germany

**Keywords:** neurodevelopmental disorders, developmental delay, metabolic disorders, synaptopathies, malformations of cortical development, pathogenic variant

## Abstract

Developmental and epileptic encephalopathies (DEEs) are a group of neuropediatric diseases associated with epileptic seizures, severe delay or regression of psychomotor development, and cognitive and behavioral deficits. What sets DEEs apart is their complex interplay of epilepsy and developmental delay, often driven by genetic factors. These two aspects influence one another but can develop independently, creating diagnostic and therapeutic challenges. Intellectual disability is severe and complicates potential treatment. Pathogenic variants are found in 30–50% of patients with DEE. Many genes mutated in DEEs encode ion channels, causing current conduction disruptions known as channelopathies. Although channelopathies indeed make up a significant proportion of DEE cases, many other mechanisms have been identified: impaired neurogenesis, metabolic disorders, disruption of dendrite and axon growth, maintenance and synapse formation abnormalities —synaptopathies. Here, we review recent publications on non-channelopathies in DEE with an emphasis on the mechanisms linking epileptiform activity with intellectual disability. We focus on three major mechanisms of intellectual disability in DEE and describe several recently identified genes involved in the pathogenesis of DEE.

## 1. Introduction

About 40% of epileptic seizures in the first years of life are caused by developmental and epileptic encephalopathy (DEE) [1,2]. DEEs are a group of diseases characterized by epileptic seizures or epileptiform activity, a severe delay or regression of psychomotor development, and cognitive and behavioral deficits [3]. Seizures and developmental delay in DEEs have a common, usually genetic, etiology and affect each other but progress independently. Often, onset of epilepsy is so early that it is impossible to determine the underlying cause. Thus, the consequences for neurodevelopment in DEEs are associated with a combination of the direct effects of the genetic variant and the impact of epileptiform activity, both of which can contribute to pathogenesis to varying degrees [4,5,6,7].

Both seizures and the progression of cognitive impairment cause severe consequences, compromising the quality of life and burdening families with financial and emotional difficulties. Depending on the DEE variant, the mortality before the age of 20 can reach 25% in some syndromes, and the remaining patients suffer from mental, behavioral, and movement disorders [8,9,10]. The situation is aggravated by the limited efficiency of existing drug therapy in controlling seizures and improving the neurological condition [9]. In our days, early diagnosis is key in managing the outcome of DEE—timely detection of the disease and its etiology directly correlates with a more favorable treatment outcome and long-term prognosis [5].

Pathogenic variants are found in 30–50% of patients with DEE [11,12,13]. Next-generation sequencing technologies have significantly accelerated the identification of genetic alterations in DEE patients [14]. The identification and characterization of such variants provide insights into the molecular mechanisms of the disease. The dissection of the underlying mechanisms can provide the basis for personalized therapies that will not only alleviate the severity of attacks but also improve the cognitive outcome of affected children [15]. Equally important is to understand the genetic etiology and dissect genotype–phenotype correlations in order to facilitate diagnosis and counseling of patient families [16,17].

The literature on DEE in the last decade has focused mainly on channelopathies. Although channelopathies indeed make up a significant proportion of DEE cases with pathogenic gene variants, many others associated with DEE have been identified: these variants cause disruption of different aspects of the brain development and function such as metabolism, progenitor proliferation, neuronal migration, dendrite and axon formation and synaptogenesis. The vast majority of reviews on DEE are devoted to epilepsy and encephalopathy in DEE and often do not discuss mechanisms of cognitive impairment. Intellectual disability, however, is no less severe for patients and complicates potential treatment. Therefore, it is important to consider the mechanisms of seizures and retardation in conjunction with each other. The goal of this review is to assess recent publications on non-channelopathies in DEE with an emphasis on the mechanisms linking epilepsy with intellectual disability.

## 2. Pathogenesis of Developmental Delay and Intellectual Disability in Developmental and Epileptic Encephalopathy

Developmental and epileptic encephalopathies (DEEs) are characterized by associated neurological pathologies such as developmental delay and intellectual disability. Cognitive deficits in DEE are commonly diagnosed in infancy or early childhood [18]. Cognitive impairment in DEE is a consequence of both the underlying encephalopathy and the accompanying seizures or epileptiform activity detectable on EEG [4,5]. Prolonged neuronal hyperexcitation during seizures, regardless of the pathway, contributes to cognitive decline [19,20,21].

Normally, cognitive functions depend on the coordinated work of neural networks that ensure the effective transfer of information between different brain regions. In epileptic encephalopathies, seizures and epileptiform activity lead to chaotic discharges that disrupt this coordination and destroy the functional connections between neurons [22]. As a result of this process, the integration of sensory information, executive control, and memory is disrupted. This subsequently impairs the development of cognitive abilities. Chronic disruptions in neural synchrony exacerbate developmental delay and contribute to the formation of persistent intellectual disability [23].

Pathogenic gene variants are the main cause of intellectual disability and developmental delay in most cases. Many of these variants are associated with dysfunction of voltage-gated ion channels. Since ion channels affect the generation, propagation, and control of action potentials, such changes often also lead to epileptic activity [6,24]. Ion balance disruption causes hyperexcitation of neurons leading to a distortion of neural function and subsequently cognitive impairment [25,26,27,28]. Most DEEs are characterized by early onset. The nervous system is most vulnerable to abnormal electrical activity during early development. Therefore, ion channel dysfunctions contribute to neuronal damage or death, further exacerbating cognitive and developmental deficits [21].

In addition, some studies describe the disruptions in synaptic plasticity due to pathogenic gene variants [29]. Indeed, neuronal plasticity is a key mechanism underlying higher cognitive abilities such as memory and learning [30]. In epileptic encephalopathies, changes in the expression of genes regulating synaptic plasticity can exacerbate cognitive deficits, since neurons lose the ability to adapt to new conditions or form new connections [31]. This further highlights the complexity and multifactoriality of cognitive impairment in DEE, since ion channels, synaptic plasticity, and neural networks in general can be simultaneously involved in the DEE pathogenesis [32].

Metabolic disturbances in neurons cause severe consequences due to excessive accumulation of metabolic products, disruption of energy metabolism, and decreased inhibition. Energy imbalance and accumulated metabolites disrupt signaling between cells, contribute to neuroinflammation, or even lead to neuronal death. Epileptic activity disrupts metabolism in the focal seizure area, as well as in the neighboring regions [33].

Increased excitability can impair neuronal migration during development. It was shown that temporary activation of migrating projection neurons (PNs) in the developing cerebral cortex causes changes in metabotropic glutamate receptors transcription, premature dendritic branching, and retention of neurons in deeper cortical layers [6,34]. On the other hand, hyperpolarization of neuronal progenitors in the ventricular zone of the mouse neocortex induced changes in transcription and cell division characteristics at later stages of development: they acquired unusual morphological and molecular features. On the other hand, intermediate progenitors expressing transcription factor Tbr2 were formed prematurely. All this indicates that changes in bioelectrical activity during neurogenesis can disrupt temporal programs of neuronal differentiation, causing abnormal neuronal function [35].

Epileptic seizures and epileptiform activity damage neural networks, which are the main substrates of cognitive functions. The basis for the functioning of neural networks in the cerebral cortex and hippocampus is long-term potentiation (LTP), a process of enhancing of conduction of nerve impulses in synaptic transmission over a long period of time [36]. It plays a major role in learning, memory, and the development of sensory systems. LTP is responsible for the stable operation and strengthening of synaptic connections [37]. Chronic seizures, in turn, cause impairment of LTP [18,38].

The location of epileptic activity within the brain is a decisive factor for the cognitive outcome of seizures—damage to functional areas causes their impairment. The hippocampus is considered to be one of the most important structures in memory formation. After seizures, the pyramidal cells of the hippocampus form abnormal neural connections, which leads to impairment of long-term, short-term, and spatial memory [21,39]. The frontal lobe of the neocortex is often damaged too, resulting in impairment of logical thinking, working memory, control of emotions, and voluntary movements [20].

**Figure 1:**
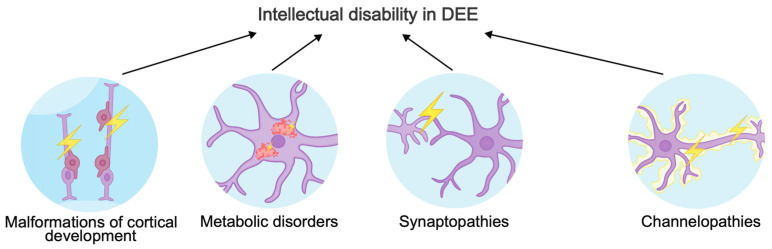
Mechanisms underlying intellectual disability in developmental and epileptic encephalopathies.

The phenotypic spectrum of gene variants causing DEE is very broad. Different versions in a single gene can cause different consequences for a protein: “gain-of-function” variants most often result in early-onset DEE, while “loss-of-function” variants lead to late-onset DEE, intellectual disability, and ASD [40]. In this review, we will focus on three major mechanisms of intellectual disability in DEE (Figure 1). In addition, we will describe new genes involved in the pathogenic molecular cascades and ignored by other reviews about DEEs.

## 3. Molecular Mechanisms Underlying Developmental and Epileptic Encephalopathy

The most frequent reasons for DEE have a genetic etiology [10,17]. The diseases are often monogenic, but oligogenic variants also occur [41,42]. According to exome sequencing and whole-genome analysis, de novo variants are the main causes, but there are also other inherited forms: autosomal recessive, dominant, and X-linked variants [12,42,43,44]. The majority of pathogenic variants are associated with channelopathies, metabolic disorders, membrane transport, and progenitor growth and proliferation during neurogenesis [45]. There is a short description of genes discussed below in Table 1.

### 3.1. Malformations of Cortical Development as a Cause of DEE

**Figure 2:**
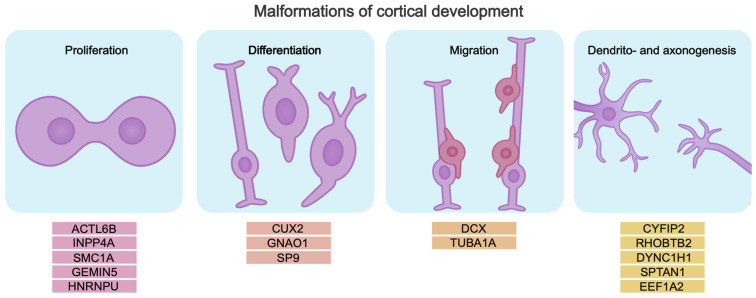
Pathogenic gene variants lead to disruption of key neurogenesis processes, which may be the cause of intellectual disability in DEE. The genes are divided into subgroups by mechanism, based on their role in the pathogenesis of intellectual disability in DEE.

Cerebral cortex development relies on correct temporal activation and inactivation of tightly regulated genetic programs that control the proliferation and differentiation of neuronal progenitors, specification, migration, and formation of neuronal circuits. All this determines the formation of a brain that functions properly. Malformations of cortical development (MCDs) are associated with impaired cerebral cortex development. Pathogenic gene variants disrupting these processes cause abnormalities in brain morphology and function. Pathogenic variants can be associated with genes encoding chromatin modifiers, transcription factors, and RNA-binding proteins that control the process of neurogenesis. Mutations in such genes cause neurodevelopmental disorders including DEEs [75,76,77,78,79,80,81] (Figure 2).

#### 3.1.1. Neuronal Progenitor Proliferation Disruption

Pathogenic variants of the neuron-specific chromatin remodeling complex (BAF), which regulate the expression of genes involved in the control of neocortical lamination, dendritic branching, and synapse formation, have been described [82,83,84,85]. Pathogenic variants of BAF, *ACTL6B*, are associated with severe forms of DEE with profound developmental delay and intellectual disability [86,87,88,89,90]. ACTL6B protein controls DNA transcription accessibility and is required for the maintenance of neuronal progenitor cell (NPC) proliferation balance. The proliferative state is maintained by the (np)BAF complex with ACTL6A during neurogenesis, whereas the differentiation of NPCs into mature postmitotic neurons requires a switch from ACTL6A to ACTL6B in the (n)BAF complex [83,89,91,92]. The most common pathogenic variants have been found in the actin-like domains of the protein, causing loss of protein function and disruption of the BAF complex assembly. They cause dysregulation of genes associated with the self-renewal of neuronal progenitors, causing abnormal cytoarchitecture of the neocortex and subsequently intellectual disability. Thus, among the clinical signs of *ACTL6B* variants are intellectual disability, developmental delay, lack of speech, hypomyelination, agenesis of the corpus callosum, and severe epilepsy [86,88,89,93,94,95,96]. Neuronal cell culture experiments demonstrated disrupted synapse formation, supporting the important influence of *ACTL6B* in neuronal development [89]. Thus, loss of *ACTL6B* function reduces the ability of neurons to form synaptic connections and leads to impaired neuronal differentiation, which plays a critical role in DEE pathology and intellectual disability.

Pathogenic variants of *INPP4A* are associated with disruption of intracellular signaling pathways. Biallelic truncated variants cause a spectrum of neurodevelopmental disorders from mild intellectual disability to DEE and microcephaly [56]. The *INPP4A* gene encodes the enzyme inositol polyphosphate-4-phosphatase, which is involved in the metabolism of inositol in phosphoinositide signaling pathways and regulates vesicle transport, which is crucial for neuronal function. Mouse models demonstrate elevated neuronal death due to defective proliferation [97,98,99,100]. Moreover, mice with a pathogenic variant of *Inpp4a* have defects in the development of the striatum, which is important for normal motor and cognitive behavior. In addition, in neuronal cultures, *INPP4A* has been shown to regulate NMDAR synaptic localization, protect neurons from excitotoxic death, and thereby maintain the functional integrity of the brain [101]. In sum, *INPP4A* is critical for the development of the nervous system, since the product of this gene influences the functioning of multiple signaling pathways, maintains cellular homeostasis and neurogenesis, and plays a role in cell proliferation and suppression of glutamate excitotoxicity [101,102,103,104].

The next interesting player in neurogenesis disorders is *SMC1A*. This gene encodes a component of the cohesin complex, which is involved in chromosome segregation during replication, DNA repair, and transcriptional regulation [105,106,107,108]. Pathogenic variants of this gene can lead to Cornelia de Lange syndrome with specific developmental delay and can also induce early DEE. Moreover, DEE associated with *SMC1A* is characterized by global developmental delay and occurs exclusively in women, due to the probable male lethality [106,109,110].

Changes in this gene in embryonic brain stem cells caused decreased DNA loops, loss of cohesin on promoters and enhancers, changes in gene expression, and proliferation defects. Supposedly the defects in *SMC1A* lead to chromosomal instability and gene expression disorders in the early stages of brain development, contributing to the neurodevelopmental pathologies [105,106,107,108,111].

Impaired neurogenesis may also be caused by other mechanisms. For example, the formation of the correct pool of mRNA isoforms is necessary for neuronal progenitors to exit the cell cycle. The disruption of RNA splicing programs during early brain development plays an important role in the etiology of NDDs [112,113,114,115]. The gene *GEMIN5* encodes a multifunctional protein involved in the assembly of small nuclear ribonucleoproteins (snRNPs), the regulation of pre-mRNA splicing, and, in general, translation [116,117,118,119,120,121,122,123,124]. Defects in *GEMIN5* are associated with cerebellar atrophy, intellectual disability, movement disorders, and early infantile developmental epileptic encephalopathy (EIDEE) [3,125,126]. Pathogenic variants of *GEMIN5* impair the ability of GEMIN5 to interact with other proteins of the SMN complex or to bind snRNA. “Loss-of-function” variants of *GEMIN5* are the most common and result in impaired translation and decreased binding of the internal ribosome entry site (IRES), which causes defects in the expression of genes essential for nervous system development [122,124,127,128,129]. “Loss-of-function” variants of *GEMIN5* increase the activity of pathways associated with postsynaptic membrane signaling and neurotransmitter secretion and decrease the activity of pathways associated with cell development, the extracellular matrix, and nuclear transport [129]. *Gemin5* is thought to play a critical role in early mammalian development. Homozygous knockout models are embryonic lethal [130,131]. Biallelic variants in *GEMIN5* are also known to cause developmental delay, motor dysfunction, and cerebellar atrophy. This is likely due to decreased levels of snRNP complex assembly proteins and defects in target RNA regulation [129].

The *HNRNPU* gene is one of the genes regulating RNA processing. It encodes heterogeneous nuclear ribonucleoprotein U (hnRNP U), a protein that plays a key role in maintaining the three-dimensional genome structure [81,132,133,134,135,136,137,138]. *HNRNPU* is widely expressed in the brain, especially in the cortex, hippocampus, and cerebellum [139]. Pathogenic variants are recognized as causes of NDD, intellectual disability, ASD, and early DEE (EIEE54) [55,114,115,140,141]. *HNRNPU*-associated developmental pathologies are mostly caused by loss-of-function defects, which lead to a spectrum of neural pathologies: abnormal neuronal migration, enlargement of the lateral ventricles, and defects in the formation of the corpus callosum [81,142,143,144]. Mouse models with *Hnrnpu* haploinsufficiency demonstrate abnormalities in brain organization and pathologies of neuronal projection and migration pathways. Since all reported human variants are heterozygous, homozygous *HNRNPU* ones probably lead to prenatal death in humans, similar to mice [80,81,115,137,145]. *HNRNPU* haploinsufficiency supposedly prevents neuronal progenitors from exiting the cell cycle and initiating differentiation, disrupting the neuronal developmental trajectory. This leads to impaired neural development and causes a spectrum of neurological disorders [115,146,147].

#### 3.1.2. Neuronal Differentiation Disruption

Another gene implicated in DEE known to be important for neurogenesis is *CUX2* [148]. *CUX2* encodes a transcription factor regulating the proliferation of neuronal progenitors in the subventricular zone (SVZ) and their differentiation and exit from the cell cycle. *CUX2* is expressed late in the cell cycle, before the final mitosis of neuronal progenitors in the SVZ [149,150,151]. *Cux-2* with *Cux-1* together are early markers of neuronal differentiation: the *Cux1* gene is involved in proliferation, and the *Cux2* gene controls cell type specification and neuronal differentiation. It is also known that *Cux* gene expression is required for the differentiation and development of interneurons [149,150,152,153,154]. Delayed *CUX2* expression can lead to abnormal cell cycle exit, causing defects in corticogenesis and subsequent neurodevelopmental pathologies [149,150,151].

Another group of genes whose pathogenic variants cause intellectual disability is involved in intracellular signaling cascades. Thus, in recent years, de novo variants of the G-protein subunits have been identified. For example, pathogenic variants of the *GNAO1* gene are associated with severe neurological syndromes, ranging from developmental delay with movement disorders to EIEE [155,156,157,158,159,160,161,162]. *GNAO1* encodes the alpha subunit of Gα, a heterotrimeric G protein that regulates intracellular signaling. The highest level of *GNAO1* is observed in the growth cone of differentiating neurons. Gα is responsible for molecular signaling that directs the growth cone navigation lead by external signals. This process is key for correct neural circuit formation [162,163,164,165]. Defects in Gα disrupt the protein’s ability to bind and hydrolyze GTP, reduce interactions with partner proteins, and cause a loss of the protein in the cytoplasmic membrane. Because of a key role in multiple neuronal signaling systems, Gα variants cause various defects in development. For example, they lead to impaired neurite growth and extension [166,167,168,169,170]. Mouse models of *Gnao1* exhibited early postnatal lethality, decreased numbers of cortical neuronal progenitors, and enlarged lateral ventricles [171]. In contrast, patients with impaired *GNAO1* had decreased levels of neurogenesis genes, increased expression of astrocyte markers, differentiation defects, and abnormal neural network formation. They had low intracellular free calcium concentrations and impaired neurotransmitter responsiveness. Thus, pathogenic *GNAO1* variants impair neural communication [172].

The next example is the *SP9* gene, which encodes a transcription factor of the Sp/KLF family, which is necessary for the regulation of gene expression in neurogenesis. *SP9* is expressed during embryogenesis in the cerebral cortex and basal ganglia, where it is necessary for the correct differentiation, migration of neurons, and the formation of neural circuits [59,173]. Several studies have reported two main types of NDD caused by defects in the *SP9* gene. A loss of function in the third C2H2 binding domain results in developmental delay, epilepsy, and autistic disorders, while changes in the second domain result in EE [59,174]. *SP9* is involved in the development of the corticospinal tract and tangential migration of GABAergic neurons. The gene also plays an important role in the proliferation and differentiation of striatopallidal projection neurons. Without *SP9*, cortical interneurons do not migrate to the cortex or striatum. *Sp9*-knockout animal models exhibit reduced cortical interneuron density, abnormal network organization, and defective axonal growth. Thus, *Sp9* knockout results in cognitive and motor impairments similar to those seen in patients with DEE [59]. It appears that loss-of-function *SP9* disrupts the transcriptional control of genes critical for corticogenesis, causing neuronal mislocalization, defective circuit formation, and altered synaptic plasticity [59,175].

#### 3.1.3. Neuronal Migration Disorders

Disruption of neuron migration during brain development may be the cause of DEE [160,176]. Appropriate regulation of cytoskeletal dynamics, particularly microtubules, is essential for neuronal migration [177]. Tubulins play an important role in this process, being essential for mitosis, axonal transport, neuronal migration, and synapse formation [178,179,180]. One of these genes, *TUBA1A*, encodes the α-tubulin isotype, which is highly expressed in postmitotic neuronal cells but absent in neuronal precursors [181,182,183,184]. α-tubulin forms heterodimers with β-tubulin to form microtubule polymers. Microtubule dysfunction can lead to various disorders of neural development referred to as tubulinopathies [180,185,186].

Pathogenic variants of *TUBA1A* are the main genetic cause of lissencephaly and can also lead to microcephaly, corpus callosum abnormalities, gray matter heterotypes, and DEE [61,187,188,189]. Variants that cause a loss of function (LoF) of *TUBA1A* result in a lack of tubulin in cells, as these variants are unable to polymerize microtubules. On the other hand, gain-of-function (GoF) variants are able to form microtubules but are unable to interact with dynein [180,185,190,191].

*Tuba1a* mutants have impaired radial neuronal migration. Mouse models of the pathogenic Tuba1a variant exhibit perinatal mortality in the homozygous state and severe brain malformations by E16.5. These mice show a decrease in the thickness of *CTIP2+* and *PAX6+* neuronal layers and apoptotic neuron death. The severe phenotype of neurodevelopment is associated with a decrease in postmitotic and apical neuronal precursors [180,192,193].

Another key gene for neuron migration is *DCX*. It encodes the doublecortin protein, which is involved in organizing microtubules during neuronal differentiation and the migration of interneurons to the cerebral cortex [194,195,196,197,198,199]. Pathogenic variants of *DCX* disrupt the structure of the N- and C-terminal regions of the protein, which are necessary for binding to microtubules and unpolymerized tubulin. These changes in the DCX protein prevent neurons from interacting properly, leading to impaired neuron migration and defects in the architecture of the developing brain cortex [195,200,201].

These pathogenic variants have been clinically associated with severe brain malformations, subcortical band heterotopia, lissencephaly, intellectual disability, epilepsy, and DEE [195,197,202]. The most severe variants of the phenotype are associated with de novo frameshift variants, while missense variants cause milder developmental defects. *DCX* is located on the X chromosome. Therefore, the most severe consequences of pathogenic variants of this gene occur in males, manifesting as severe MCD, lissencephaly, developmental delay, intellectual disability, and seizures. Females, on the other hand, have a milder phenotype in the form of heterotopia [195,203,204,205].

#### 3.1.4. Dendrito- and Axonogenesis Disorders

Neuronal morphogenesis which includes the formation of dendritic trees and axons, depends on the action of multiple molecules that control cytoskeleton structure and maintenance. One such factor is the *CYFIP2* gene, which plays an important role in regulating the actin cytoskeleton via the WAVE complex [206]. When the small Rho GTPase Rac1 binds to the CYFIP2 protein, the WAVE complex is activated, and it interacts with Arp2/3 [207]. This interaction promotes actin filament polymerization and maintains polymerization/depolymerization dynamics required for neurite outgrowth and branching [208]. Defects in CYFIP2 disrupt this process, leading to actin filament destabilization and impaired outgrowth [209]. This is manifested by a reduced ability of neurons to form leaf-like lamellipodia and synaptic contacts, which entails defects in synaptic plasticity and impaired neuronal migration [210]. In patients, a pathogenic variant of *CYFIP2* leads to severe DEE, psychomotor delay, intellectual impairment, hypotonia, and behavioral disorders and may be associated with fragile X syndrome [211,212].

*SPTAN1* is another gene important for maintaining the structural integrity of neurons too. *SPTAN1* encodes the spectrin αII protein, which is also involved in the actin organization and membrane structure stabilization. *SPTAN1* ensures the structural integrity of the cytoskeleton and the normal functioning of neurons [213]. Spectrin αII binds to actin filaments, forming a supporting network under the cell membrane, which is important for maintaining the mechanical stability of membranes and synaptic plasticity [60]. This protein is also necessary for the assembly of nodes of Ranvier [214]. Pathogenic variants of *SPTAN1* result in axonal defects and disrupted cellular architecture, leading to epilepsy, developmental delay, ASD, microcephaly, spastic paraplegia, and West syndrome [213,215,216].

Cytoskeletal dynamics is also regulated by the *RHOBTB2* gene, which encodes a protein of the Rho-type GTPase family. *RHOBTB2* is involved in the regulation of cytoskeletal dynamics, cell migration, and vesicular transport, influencing cell differentiation and apoptosis [217]. Interaction of RhoBTB with the Cullin3 protein, which is part of the ubiquitin–proteasome complex, can regulate the levels of specific proteins required for normal dendritic development and synaptic plasticity. In the context of RHOBTB2, the association with Cullin3 suggests that missense variants may disrupt the degradation machinery, affecting the stability of proteins required for normal dendritic development and neuronal function. *RHOBTB2* has an important role in cell cycle control, participating in the regulation of cellular differentiation and apoptosis [218,219]. Knockout of *RhoBTB* in Drosophila dendritic neurons highlighted the critical role of the formation of dendritic architecture, decreasing the number of dendritic branches. Missense variants in the coding region of the BTB domain of RHOBTB2 are associated with DEE, indicating importance in neuronal development and possibly in the regulation of dendritogenesis [220]. However, the precise molecular mechanisms linking missense variants with neurodevelopment remain poorly understood, requiring further studies to characterize their role in neuropathology.

*The DYNC1H1* gene regulates cytoskeleton functions too. It contains the cytoplasmic dynein heavy chain, which mediates the binding of dynein complexes to microtubules [221]. This process is critical for maintaining neuronal homeostasis and delivering key components involved in synaptic activity, such as neurotransmitter receptors, synaptic vesicle precursors, and others [222,223]. Disruptions in *DYNC1H1* function can lead to defects in protein folding and microtubule bundling [224]. Patients with pathological variants of the *DYNC1H1* exhibit neurodevelopmental delay, DEE, and, in some cases, abnormal brain morphology, including microcephaly and other phenotypes [221].

Impaired inhibitory neuron function in DEE may be due to decreased levels of the Caspr2 protein, encoded by the *CNTNAP2* gene [225]. This gene encodes contactin-associated protein-like 2, a member of the neurexin family—cell adhesion molecules involved in the formation of synaptic contacts [226,227]. *CNTNAP2* is necessary for myelination, axon guidance, organization of dendritic branching, and spine formation, and therefore, it controls the formation of neural networks in general [228]. *CNTNAP2* deficiency causes increased neuronal excitability [229]. In particular, recessive variants in the *CNTNAP2* gene affect the levels and functions of *GluA1*, a subunit of AMPA receptors regulating excitatory synaptic transmission [230]. Disruption of *CNTNAP2* leads to altered expression, surface localization, and endocytosis of *GluA1*, attenuating synaptic plasticity and modulating the activity of calcium-dependent signaling pathways [231]. Patients with a recessive variant in the *CNTNAP2* gene demonstrated cognitive impairment, language disorders, seizures, and focal cortical dysplasia epilepsy syndrome (CDFE) and also had a decrease in the number of GABAergic interneurons and neuronal migration abnormalities, indicating profound defects in the formation and functioning of neural networks [47].

The *EEF1A2* gene plays an important role in the translation and organization of the neuronal cytoskeleton. It encodes eukaryotic translation elongation factor 1A2, which affects the process of protein synthesis. EEF1A2 binds to amino acids and tRNAs and participates in the transfer of tRNA to the A-site of the ribosome, which is necessary for the elongation of the polypeptide chain during translation. Pathogenic variants of *EEF1A2* are associated with DEE, developmental delay, and microcephaly [232] because they disrupt translation (due to increased tRNA binding), reducing the translation velocity. This affects the morphological development of cortical neurons. Pathogenic *EEF1A2* has lower actin-binding activity. Thus, EEF1A2 has two functions: translation regulation and organization of the neuronal cytoskeleton [233]. When *EEF1A2* was knocked out in human glioblastoma cells, the process of cell proliferation and differentiation was impaired [234].

In summary, as the cerebral cortex develops, multiple molecular pathways interact to produce a complex neuronal network making up the cerebral cortex. The disruption of any of these pathways can lead to serious pathologic conditions. This, in turn, can cause the development of intense and sometimes multifocal epileptic activity associated with DEE.

### 3.2. Synaptopathies—Synaptic Transmission Disorders

**Figure 3:**
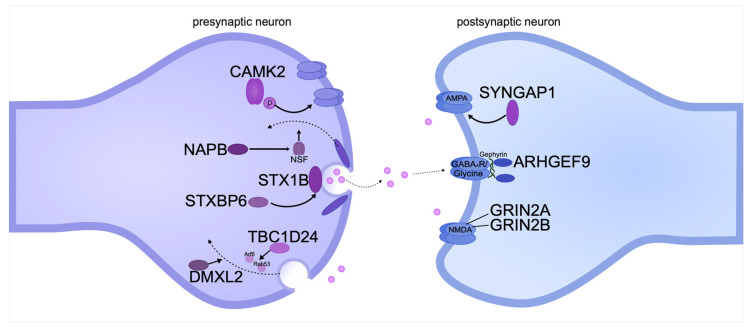
Pathogenic gene variants encoding pre- and postsynaptic transmembrane proteins can cause intellectual disability in DEE. The diagram shows the location of proteins relative to the synaptic cleft and the functions they perform. The proteins are systematized based on recent publications on DEE.

Pathogenic gene variants affecting pre- and postsynaptic transmembrane proteins can lead to DEE both directly and indirectly. Pre- and postsynaptic membranes are involved in the transport of synaptic vesicles in axons and action potential initiation in dendrites (Figure 3).

The SNARE protein complex plays an important role in the presynaptic membrane [235]. One of the members of the complex is syntaxin-1B, which is encoded by the *STX1B* gene. The main role of the protein is to anchor synaptic vesicles to the presynaptic membrane [236]. Syntaxin-1B has two conformations: open, which is necessary for the formation of the SNARE complex, and closed, which initiates the vesicle fusion reaction [237,238]. De novo *STX1B* pathogenic variants are clinically associated with DEE and generalized epilepsy with febrile seizures [67]. Most often, missense variants are loss-of-function variants of the open conformation of the protein, resulting in disruption of the assembly of the complex and vesicle transport. Pathogenic variants of the closed conformation of the protein lead to the disruption of protein–protein interactions and normal fusion of presynaptic vesicles [239]. Mice with a gene knockout of *Stx1b* exhibit severe seizures and premature death associated with dysfunction of neurotransmitter release at GABA and glutamatergic synapses [240]. In addition to STX1B, other regulatory proteins, such as the product of the *STXBP6* gene, are involved in maintaining the fidelity of vesicle–membrane fusion processes. *STXBP6*, encoding syntaxin-binding protein 6, also known as amysin, is involved in modulating syntaxin activity and controlling membrane interactions, which is necessary for the normal functioning of the synaptic apparatus, namely the movement of neuronal vesicles [241]. A patient with epileptic encephalopathy and autism spectrum disorder (ASD) was found to have a truncated variant of the protein encoded by the *STXBP6* gene [242]. Mice with a deletion of this gene had reduced body weight, which is also one of the phenotypes in some ASD patients. However cognitive skills were not impaired in these mice [243].

Neurotransmitter release occurs through Ca(2+)-induced synaptic vesicle fusion mediated by the SNARE complex [244]. The SNARE complex is associated with the βSNAP protein, which is a product of the *NAPB* gene. βSNAP is one of the cofactors of NSF-ATPase, which is essential for synaptic transmission, since this enzyme is involved in the disassembly and utilization of SNARE complex proteins [245]. Whole-exome sequencing of three siblings with severe intellectual disability and DEE revealed a seven-base-pair deletion in the *NAPB* gene, resulting in a 46% truncation of the protein. The children developed epileptic seizures before 6 months of age and severe developmental regression by 2 years of age [246]. Recently, whole-exome sequencing of an Arab-Palestinian consanguineous family of three identical twins diagnosed with Cohen syndrome was performed. The twins suffered from early-onset epileptic encephalopathy, autism, and intellectual disability. Analysis of the sequencing data identified a pathogenic variant affecting the splice site of the *NAPB* gene [247]. Mice with reduced *βSnap* expression showed epileptic seizures, followed by ataxia and, in some cases, death [245].

Among the postsynaptic membrane proteins, pathogenic variants of *SYNGAP1* are most frequently associated with DEE [248]. This protein is a key mediator in the RAS signaling cascade activated by the NMDA receptor. During LTP, *SYNGAP1* activates RAS-GTPase (SynGAP) in glutamatergic neurons, resulting in the insertion of AMPA receptors and an increase in synaptic surface area [249,250,251]. *SYNGAP1* pathogenic variants affect glutamatergic synapses and enhance glutamate receptor activity, increasing the probability of epileptogenesis [252]. *SYNGAP1* mRNA has multiple alternatively spliced variants encoding different protein isoforms that differ in structure, function, and temporal expression. Four C-terminal isoforms have been identified: α1, α2, β, and γ. The β isoform is expressed early in postnatal development, while α2 is expressed at higher levels in the mature brain [253,254]. This explains the differences in phenotypic severity; for example, nonsense-mediated decay caused by defects in early isoforms leads to complete loss of the gene product. A milder outcome of SYNGAP1-DEE is observed in patients with splice site variants in exons 1 through 4 [68].

Another gene associated with synaptopathy is *TBC1D24*. This gene encodes a protein that activates the small GTPases Arf6 and Rab35, which act antagonistically. They are required for membrane transport at synapses, as well as between the plasma membrane and endocytic compartments [255]. *TBC1D24* has a broad expression pattern and is found in all layers of the cerebral cortex and the hippocampus. Pathogenic variants of *TBC1D24* cause dysregulation of synaptic vesicles, which causes excessive neurotransmission. In addition, it interferes with the normal disposal of defective proteins through endosomal pathways, leading to their accumulation and neuronal dysfunction. These alterations contribute to the development of a wide range of epileptic phenotypes and other neurodevelopmental disorders in patients [69]. Knockout of the *Tbc1d24* gene in rat primary cortical neurons revealed impaired axon initial segment formation and neuronal excitability. This phenotype was associated with increased activation of the GTPase Arf6, which is required for axon specification and neurite extension [256].

The *DMXL2* gene encodes a large protein that is associated with vesicular transport and plays a key role in the regulation of synaptic transmission. Disruptions in the function of the DMXL2 protein can lead to disruptions in synaptic endocytosis and vesicle recycling. This is due to the fact that DMXL2 regulates the acidification of intracellular compartments via the vacuolar proton pump (V-ATPase) [257]. In addition, the DMXL2 protein acts as a modulator of the Notch signaling pathway and is required for chromatin recruitment of Notch-dependent transcription factors [258]. Pathogenic variants of *DMXL2* can disrupt these processes, which leads to an imbalance of excitation/inhibition in the nervous system, causing neuronal hyperexcitability. This, in turn, is associated with the development of epileptic seizures and severe developmental delay, characteristic of DEE [6]. The neuronal hyperexcitability underlying DEE may also be associated with dysfunction of glutamate receptors. In particular, the *GRIN2A* and *GRIN2B* genes encode subunits of NMDA (N-methyl-D-aspartate) receptors, which are subtypes of glutamate receptors. They play a key role in synaptic plasticity, learning, and memory. These receptors control the entry of calcium, sodium, and potassium ions across the neuronal membrane, which is necessary for the transmission of excitatory signals in the brain [65,259]. Increased activity of NMDA receptors leads to excessive calcium influx into cells, which can cause neuronal hyperactivity and, as a result, neuronal death [260]. There are multiple rare variants of *GRIN2A* and *GRIN2B* genes associated with neurological diseases. Currently, 304 variants that cause DEE have been reported in *GRIN2A*, and 273 variants that cause DEE have been reported in *GRIN2B* (ClinVar) [261]. The phenotypic manifestations in these genes have been studied in detail in a number of clinical studies. Patients with pathogenic variants of *GRIN2A/GRIN2B* exhibit severe forms of epileptic encephalopathy, accompanied by delayed motor and cognitive development. These clinical manifestations correlate with disturbances observed at the level of synaptic transmission and neuronal activity [262].

The *ARHGEF9* gene encodes the protein collibostin (Cb), which regulates the actin cytoskeleton dynamics and synaptic activity through activation of Rho GTPases, in particular Cdc42 [263]. It interacts directly with the scaffold protein gephyrin and is required for the formation of gephyrin-dependent GABA A clusters on the postsynaptic membrane [264]. Cb interaction occurs due to the presence of the SH3 domain, which binds to the large intracellular loop of the α2 subunit of GABA A receptors [265]. Point mutations in *ARHGEF9* disrupt inhibitory synaptic transmission through interaction with GABA and glycine receptors, which leads to neuronal hyperexcitability and cognitive impairment. It is associated with the development of epilepsy, ASD, intellectual disability, and, in some cases, certain facial dysmorphism [62,147].

Ca 2+/calmodulin-dependent protein kinase II (CAMK2) is one of the most important enzymes in synaptic plasticity and memory formation [266]. The protein consists of two predominant subunits, alpha (CAMK2A) and beta (CAMK2B), that are highly homologous to each other and can probably substitute each other’s functions when one is inactivated [267]. Pathogenic variants of *CAMK2A* or *CAMK2B* cause intellectual disability, ASD, and DEE in humans [268]. The CAMK2 enzyme is part of the Ca-dependent signaling pathway and phosphorylates various substrates responsible for LTP [269]. When activated, CAMK2A exerts significant effects on dendritic spines and postsynaptic density by interacting with enzyme-associated proteins, particularly the GluN2B subunits of the NMDA receptor [270]. When the CaMK2A autophosphorylation site is disrupted in mice, defects in spatial learning and memory are observed [271].

Thus, synaptopathies are one of the key mechanisms underlying epileptic encephalopathies and neurodevelopmental disorders in general. Disturbances in synaptic plasticity lead to dysregulation of neural connections, which causes epileptic activity in the brain and significant cognitive and motor deficits. This undoubtedly emphasizes the importance of studying synaptopathies for understanding the pathogenesis of epileptic disorders and associated developmental delays [31].

### 3.3. Metabolic Disorders

The mammalian brain has a high energy demand. Most of the energy is utilized for the activation of action potentials and synaptic transmission. It is provided by glycolysis and mitochondrial respiration. On the other hand, energy demand during neurogenesis is extremely high as well. It is not surprising therefore that abnormal bioenergetics and mitochondrial dysfunction in neurons cause cognitive disorders [272].

One such disorder is caused by pathogenic variants of *HK1*, which encodes hexokinase HK1. This enzyme carries out ATP-dependent phosphorylation of glucose to glucose-6-phosphate (G6P) in glycolysis [273]. *HK1* is predominantly expressed in neurons and astrocytes in the brain, and gene dysfunction has been associated with multiple developmental disabilities, including neurodevelopmental disorders (NDDs) and DEE [72,273].

**Figure 4:**
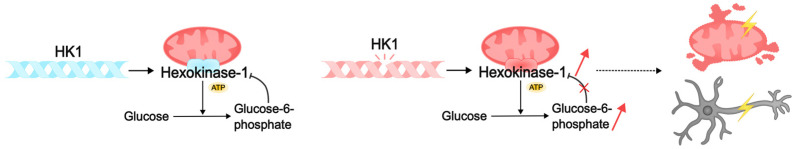
Pathogenic variants of *HK1* can lead to intellectual disability by disrupting cellular metabolism. Normally, HK1 hexokinase catalyzes the phosphorylation of glucose to glucose-6-phosphate (G6P), which binds to the enzyme domains. Competitive inhibition with ATP blocks kinase activity. Pathogenic *HK1* disrupts the reverse binding of G6P to the enzyme domains. As a result, the kinase continues to constitutively phosphorylate glucose, which leads to the accumulation of metabolites, damage to mitochondria, and death of neurons. Lack of energy and dysfunction of neural networks can subsequently lead to intellectual disability in DEE.

Pathogenic variants of *HK1* can cause intellectual disability through several mechanisms related to the essential functions of hexokinase in cellular metabolism and neuronal activity. HK1 consists of two symmetrical monomers that contain an alpha-helix-linked regulatory N-terminal domain and a catalytic C-terminal domain [274,275]. The phosphorylation product of this hexokinase, G6P, binds to both domains of the enzyme, resulting in competitive inhibition of ATP binding and inhibition of kinase activity [276,277]. This process is disrupted by missense variants in the alpha helix and regulatory domain of the enzyme, which makes binding of G6P to the HK1 domains impossible, and the enzyme loses its ability to self-regulate. Such defects result in HK1 “gain of function”: the enzyme continues to constitutively phosphorylate glucose, leading to the accumulation of metabolites and mitochondrial damage [278]. It is thought to result in the accumulation of misfolded proteins, endoplasmic reticulum stress, mitochondrial dysfunction, apoptosis, and cell death [72,279]. This may cause neuronal loss in brain regions responsible for learning, memory, and cognition, such as the hippocampus and prefrontal cortex. Pathogenic variants of HK1 reduce energy availability in brain cells, and energy deficiency leads to impaired neuronal activity. This, in turn, may lead to defects in the formation of neural networks during critical periods of development and, consequently, cognitive impairment and intellectual disability [6,72] (Figure 4).

Other pathogenic variants, such as those causing membrane transporter dysfunction, can also lead to disruption of neuronal bioenergetics. The *SLC25A12* gene encodes the mitochondrial aspartate–glutamate transporter (AGC1/Aralar), a component of the malate–aspartate shuttle (MAS), mainly expressed in the nervous system and muscles. This transporter carries out the antiport of cytosolic glutamate and protons in exchange for intramitochondrial aspartate. MAS function is necessary to maintain the redox balance between cytosolic glycolysis and mitochondrial respiration and ensures ATP synthesis, which is important for neurons, which have high energy requirements [280,281,282,283]. Pathogenic variants of *SLC25A12* result in AGC1 deficiency, which causes infantile epileptic encephalopathy with global psychomotor retardation and brain hypomyelination [284]. Pathogenic variants result in the disruption of the transporter gating, which limits conformational changes in the protein for substrate release in the mitochondrial matrix and impairs cellular metabolic activity [285,286,287,288]. Studies in *Agc1*-knockout mouse models demonstrate decreased cellular respiration in the brain, decreased aspartate levels, and impaired glutamate metabolism. As a result, neurons lacking *AGC1* are unable to maintain normal metabolic activity [282,288]. *AGC1* plays a central role in neuronal bioenergetics, and since neuronal growth and differentiation require increased energy production, this protein is very important during neurogenesis [272,289].

**Figure 5:**
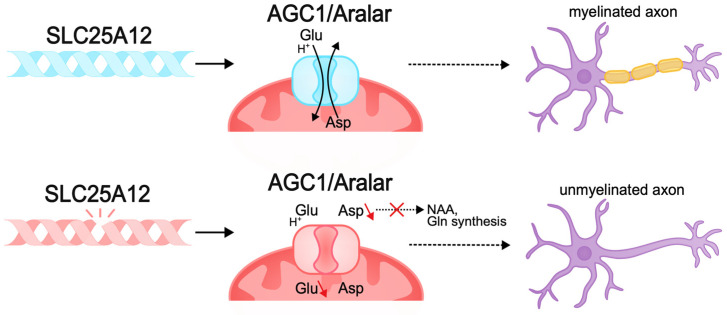
Pathogenic variants of *SLC25A12* can lead to the disruption of neuronal bioenergetics and axonal myelination. The *SLC25A12* gene encodes the mitochondrial aspartate–glutamate transporter (AGC1/Aralar). Pathogenic variants of *SLC25A12* lead to disruption of the functioning of the transporter gate and the inability to antiport aspartate and glutamate. The lack of aspartate in the nervous system causes a deficiency in the biosynthesis of N-acetylaspartate (NAA), which is necessary for the synthesis of myelin and myelination of axons. Decreased myelination disrupts the normal development of axons and their ability to transmit signals, which may be the cause of intellectual disability in DEE.

In addition, *AGC1* pathologies in the nervous system lead to aspartate deficiency and limited biosynthesis of N-acetylaspartate (NAA), which is necessary for myelin synthesis. Reduced myelination disrupts normal axon development and the ability of axons to transmit signals, causing pathologies of neurotransmission, in particular glutamatergic, which explains the intellectual deficit observed in patients [70,290,291,292] (Figure 5).

Mouse models of pathologies of this transporter have pronounced hypomyelination, as well as impaired development of cortical axons and postnatal development of cortico-hippocampal neurons [282,293,294,295,296]. Thus, pathogenic variants of *SLC25A12* lead to disruptions in neuronal function and the development of severe forms of epileptic encephalopathy with concomitant intellectual deficit, which is associated with disruptions of corticogenesis, myelination, and glutamatergic transmission due to metabolic disorder.

There are cases of intellectual disability caused by protein glycosylation defects. This post-translational modification of proteins plays an important role in many intracellular processes, including synaptic plasticity. Animal models of glycosylation disorders in the nervous system demonstrate synaptogenesis disorders, hippocampal developmental abnormalities, and intellectual disability [297,298,299,300]. One of the genes involved in the control of glycosylation is *ALG13*. The product of this gene is involved in post-translational modification of proteins by N-glycosylation, and gene expression is predominantly observed in neurons of the cerebral cortex and hippocampus [301]. Pathogenic variants of *ALG13* lead to congenital disorders of glycosylation and DEE. They are characterized by global developmental delay with regression, hypotonia, and movement disorders. They are mainly diagnosed in females [302,303,304]. Inside the cell, ALG13 forms a heterodimeric complex with the ALG14 protein, which performs an auxiliary function for anchoring ALG13 to the endoplasmic reticulum membrane. Together, they form a functional glycosyltransferase UDP-GlcNAc, which transfers N-acetylglucosamine to asparagine residues of proteins [305,306,307,308,309]. This process is necessary for the correct folding of proteins and the formation of functional glycoproteins, which ensures their stability, sorting, and transport, and is also important for the implementation of intercellular interactions [301,310,311,312]. The ALG13 protein has several isoforms: long (ALG13-is1) and short (ALG13is2). These isoforms are identical in the catalytic domain of the N-terminal region but are significantly different in the C-terminal region, the part of the protein responsible for the transport of proteins to the endoplasmic reticulum. Pathologies are caused by mutations in both the catalytic and C-terminal domains, which disrupt the activity of the protein, its interactions with the endoplasmic reticulum membrane, and the ability to glycosylate proteins. Defects in the C-terminal region of the long isoform of ALG13 are known to cause developmental and epileptic encephalopathy, intellectual disability, and type I glycosylation disorders [308,313,314,315]. The *Alg13KO* mouse model exhibited cognitive deficits, decreased dendritic complexity and length, and dendritic spine density in the hippocampus. It is likely that cognitive decline in *ALG13* pathology is caused by the failure to form correct synaptic connections [316,317,318,319]. Furthermore, *ALG13* loss was found to be characterized by neuronal death and reactive astrogliosis and may reduce inhibitory synaptic transmission by regulating the transcription of the GABA A R α2 subunit, which aggravates synaptic plasticity pathologies [301]. Collectively, pathogenic variants of *ALG13* cause profound cognitive impairment, developmental delay, and a severe DEE phenotype due to glycosylation and neuronal plasticity disorders.

Other examples of glycosylation disorders are pathogenic variants of *ST3GAL3*, which encodes the Golgi transmembrane enzyme sigleosyltransferase ST3Gal-III. This enzyme catalyzes the transfer of sialic acid to galactose in gangliosides and glycoproteins. Sialoglycans are critical for the nervous system, as they are required for normal neuronal function, intercellular communication, myelination, and synaptic plasticity [320,321,322,323,324]. Disruptions in the ST3Gal-III enzyme result in decreased levels of sialoglycans, which impair nervous system function, affecting cognitive development and learning ability. Sialoglycan deficiency disrupts the stability and function of membrane proteins, which interferes with normal neuronal signaling [325,326].

*ST3GAL3* loss-of-function variants result in West syndrome, a DEE syndrome with developmental regression and intellectual disability, and severe nonsyndromic autosomal recessive intellectual disability (NSARID) [324,325,327,328,329].

**Figure 6:**
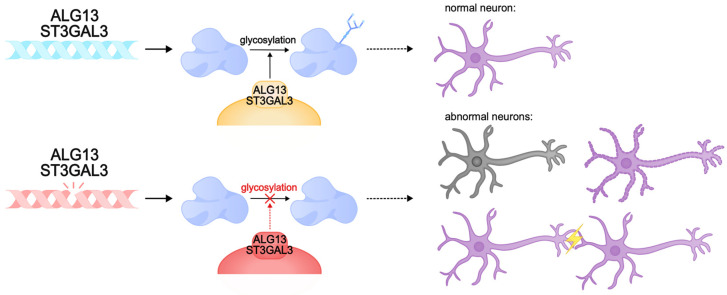
Pathogenic variants of *ALG13* and *ST3GAL3* can lead to congenital glycosylation disorders. These genes encode transmembrane glycosylation enzymes that play a critical role in the nervous system. Pathogenic variants can cause abnormalities in synapse function, neuronal membrane formation, and neuronal death. These abnormalities can result in intellectual disability, delayed development, and DEE.

Studies in the *St3gal3-null* and *St3gal2/3-null* mouse models showed that gene disruptions lead to a lack of glycoprotein sialylation and, subsequently, to hypomyelination, impaired oligodendrocyte proliferation, and abnormal formation of nodes of Ranvier. Similar to the human phenotype, these mice exhibited severe cognitive deficits, decreased motor coordination, and hyperactive behavior. In addition, the lack of adequate sialylation disrupted the proper functioning of synapses, which reduced synaptic plasticity and impaired learning and memory. Taken together, pathogenic variants of *ST3GAL3* lead to glycosylation deficiency of gangliosides and glycoproteins, and subsequently to cognitive dysfunction, and patients with pathogenic *ST3GAL3* exhibit severe intellectual disability, developmental delay, and DEE [323,325] (Figure 6).

Thus, metabolic disorders in the developing nervous system are one of the mechanisms for the development of intellectual disability in DEE. Among the causes we considered, pathogenic variants of some enzymes and transporters cause defects in bioenergetics and post-translational modification, leading to synaptic transmission and myelination pathologies, neural network formation pathologies, and neuronal death. Ultimately, all this comes down to the disruption of interneuronal communication and subsequent intellectual disability.

## 4. Conclusions

Developmental and epileptic encephalopathies (DEEs) are a group of diseases characterized by epileptic seizures, interictal epileptiform activity, and severe developmental delay with cognitive deficits. These pathologies often have a common etiology and influence each other but develop in parallel and in different ways.

A genetic etiology often underlies DEEs. In the last decade, due to the development of next-generation sequencing, many research groups around the world have discovered many pathogenic variants that cause DEE. These are often monogenic disorders that either occur de novo or are inherited recessively. The most frequent variants that cause DDE, associated with channelopathies, disrupt the function of the genes that encode voltage-dependent sodium and potassium channels, such as, for example, *SCN2A* and *KCNQ2*.

However, many DEE-causing variants have been described recently whose gene products control processes other than current conductance: metabolic disorders, membrane transport, and growth and proliferation during neurogenesis. These findings demonstrate that the pathogenesis of DEE extends far beyond neuronal transmission and any disruption of the correct numbers and proportions of different types of neurons, their positioning, synaptic input and output, axonal and dendritic transport, and energy consumption can disrupt the correct excitation/inhibition balance and cause very severe consequences in brain function that will be manifested in epileptiform activity and cause intellectual disability.

The identification and detailed investigation of the genetic causes of DEE and the molecular cascades involved are important for understanding the molecular basis of pathogenesis responsible for the occurrence of these disorders. Understanding these pathways and determining the genotype–phenotype correlation can help in the diagnosis and genetic counseling of patients’ families. Although in many cases, by the time when the disease has been diagnosed, the cytoarchitecture of the brain has been terminally malformed and treatment is no longer possible, there are DEE cases where the brain structure has not been dramatically changed. Such cases could potentially be treated individually, depending on the molecular cascade affected by the pathogenic gene variant. For example, if the cause is a metabolic disorder, a replacement therapy in combination with gene therapy can be used. Gene constructs or mRNAs that would replace malfunctioning proteins can be delivered to the brain. On the other hand, if the main cause of the disease is a “gain of function” of a certain gene, a small inhibitor molecule can be identified that attenuates the hyperactivity of the gene product. A common feature and problem of many DEEs is pharmacoresistance to antiepileptic drugs. Here, animal models replicating the pathology can be used in individual cases in order to select a treatment with a combination of antiepileptic drugs.

Uncovering the molecular mechanisms of the pathogenesis of intellectual disability in DEE can become the basis for personalized therapy that will improve not only the severity of seizures, but also the cognitive outcome in affected children.

## Figures and Tables

**Table 1:** Pathogenic variants associated with intellectual disability in developmental and epileptic encephalopathies.

Mechanism	Subgroup	Gene Name	Type of Inheritance
Malformation of cortical development	Impaired differentiation and proliferation	*ACTL6B*	Autosomal recessive; autosomal dominant; de novo [46]
	Differentiation of inhibitory interneurons	*CNTNAP2*	Autosomal recessive [47]
	Impaired differentiation and proliferation	*CUX2*	Autosomal dominant; de novo [48]
	Disruption of dendrito- and axonogenesis	*CYFIP2*	Autosomal dominant; de novo [49]
	Impaired migration	*DCX*	X-linked recessive; de novo [50]
	Disruption of dendrito- and axonogenesis	*DYNC1H1*	Autosomal dominant; de novo [51]
	Disruption of dendrito- and axonogenesis	*EEF1A2*	Autosomal dominant; de novo [52]
	Impaired proliferation	*GEMIN5*	Autosomal recessive [53]
	Impaired differentiation	*GNAO1*	Autosomal dominant; de novo [54]
	Impaired differentiation and proliferation	*HNRNPU*	Autosomal dominant; de novo [55]
	Impaired proliferation	*INPP4A*	Autosomal recessive [56]
	Disruption of dendritogenesis	*RHOBTB2*	Autosomal dominant; autosomal recessive; de novo [57]
	Impaired proliferation	*SMC1A*	X-linked dominant [58]
	Impaired proliferation and migration	*SP9*	Autosomal dominant; de novo [59]
	Impaired differentiation	*SPTAN1*	Autosomal dominant; de novo [60]
	Impaired migration	*TUBA1A*	Autosomal dominant [61]
Synaptopathies	Inhibitory synaptic transmission	*ARHGEF9*	X-linked recessive; de novo [62]
	Effects on dendritic spines	*CAMK2*	Autosomal dominant; de novo [63]
	Disruptions in synaptic endocytosis	*DMXL2*	Autosomal dominant [64]
	Dysfunction of glutamate receptors	*GRIN2A/B*	Autosomal dominant; de novo [65]
	Disruption of disassembly and utilization of SNARE complex proteins	*NAPB*	Autosomal recessive [66]
	Disruption of synaptic vesicle fusion	*STX1B*	Autosomal dominant; de novo [67]
	Enhanced glutamate receptor activity	*SYNGAP1*	de novo [68]
	Dysregulation of synaptic vesicles	*TBC1D24*	Autosomal recessive [69]
Metabolic disorders	Membrane transporter dysfunction	*AGC1*	Autosomal recessive [70]
	Congenital disorders of glycosylation	*ALG13*	X-linked recessive; de novo [71]
	Accumulation of metabolites	*HK1*	Autosomal recessive; de novo [72]
	Membrane transporter dysfunction	*SLC25A12*	Autosomal recessive [73]
	Decreased levels of sialoglycans	*ST3GAL3*	de novo [74]

## Data Availability

The data presented in this study are available on request from the corresponding author.
